# Comprehensive Toxicological Evaluation and Safety Assessment of *Perilla frutescens* Leaf Extract: Repeated-Dose Oral Toxicity and Genotoxicity Studies

**DOI:** 10.3390/foods15213852

**Published:** 2026-10-28

**Authors:** Je-Ho Lee, Ki Hwan Park, Mi-Yeong An, Jong Soon Kang, Song-Kyu Park

**Affiliations:** 1 Daehan Cellpharm Co., Ltd., 116, Donggureung-ro, Guri-si 11905, Republic of Korea; leejeho1128@naver.com (J.-H.L.); miyeong1119@gmail.com (M.-Y.A.); 2 Department of Pharmacy, College of Pharmacy, Korea University, 2511, Sejong-ro, Sejong-si 30019, Republic of Korea; 3 Laboratory Animal Resource and Research Center, Korea Research Institute of Bioscience and Biotechnology, 30, Yeongudanji-ro, Ochang-eup, Cheongwon-gu, Cheongju-si 28116, Republic of Korea; brightnessd@kribb.re.kr

**Keywords:** *Perilla frutescens*, oral toxicity, genotoxicity, rosmarinic acid

## Abstract

*Perilla frutescens* var. *frutescens* leaf extract (PFE) has previously demonstrated clinical efficacy in relieving knee joint discomfort. To establish its safety profile, good laboratory practice (GLP)-compliant nonclinical evaluations were conducted in accordance with the Organization for Economic Cooperation and Development (OECD) test guidelines. In the acute oral toxicity test, the acute oral LD_50_ cutoff was estimated to be above 2000 mg/kg. In 28-day and 90-day repeated-dose oral toxicity studies of up to 5000 mg/kg/day, no mortalities, toxic signs, or adverse changes in body weight were observed. Minor hematological/biochemical shifts and organ weight increases at 5000 mg/kg/day lacked histopathological correlates and were largely reversible following a 28-day recovery period, establishing the no observed adverse effect level (NOAEL) at 5000 mg/kg/day. Furthermore, PFE showed no mutagenic, clastogenic, or aneugenic potential across the bacterial reverse mutation, in vitro chromosomal aberration, or in vivo bone marrow micronucleus assays. These results demonstrate a broad safety margin, supporting the safe application of PFE as a functional food ingredient and nutraceutical agent.

## 1. Introduction

*Perilla frutescens* (L.) Britton (Lamiaceae) is a well-known traditional medicinal herb and dietary plant that is native to and widely cultivated throughout East Asia, particularly Korea, China, and Japan [1,2,3]. *P. frutescens* can be classified into distinct infraspecific varieties on the basis of morphology and regional utilization. *P. frutescens* var. *frutescens*, commonly known as Korean perilla or *deulkkae*, is cultivated primarily for edible seed oil and leafy vegetables and contains high amounts of *α*-linolenic acid, phenolic compounds and flavonoids [4,5]. In contrast, *P. frutescens* var. *crispa*, known as *shiso* or *cha-jo-ki*, is widely used as a culinary herb or garnish and is characterized by the accumulation of volatile monoterpenoids, such as perillaldehyde, and red anthocyanin pigments [6,7]. *P. frutescens* var. *frutescens* leaves are known to exert anti-inflammatory, antioxidant, and antiallergic effects [2,8], whereas *P. frutescens* var. *crispa* has been historically noted for its bronchial-relaxing and acute anti-inflammatory activities [9,10].

In recent years, there has been a growing interest in the development of functional foods and nutraceuticals derived from natural botanical sources to manage chronic inflammatory conditions with fewer adverse effects than conventional pharmacological treatments. While *P. frutescens* has a long history of safe dietary consumption as a whole food, modern nutraceutical applications often utilize highly concentrated extracts enriched with specific bioactive marker compounds to maximize physiological benefits [8]. However, the safety profile of traditional dietary usage cannot be directly extrapolated to the long-term consumption of these concentrated botanical extracts at higher therapeutic doses [11,12]. Chronic exposure to concentrated phytochemicals may present unanticipated systemic toxicities, target organ damages, or genotoxic risks [13]. Therefore, rigorous toxicological evaluations in compliance with internationally recognized regulatory standards, such as the OECD guidelines, are strictly required to bridge the gap between traditional dietary use and safe application as a standardized functional food ingredient [14].

Recently, a standardized *P. frutescens* var. *frutescens* leaf extract (PFE) has demonstrated promising clinical efficacy in alleviating joint pain and improving functional mobility in individuals with mild knee discomfort [3]. Despite its verified clinical utility and traditional dietary consumption, a thorough toxicological evaluation in accordance with internationally recognized regulatory standards is essential to definitively establish its safety profile for therapeutic and dietary development. Therefore, the present study was conducted to comprehensively evaluate the safety of PFE through good laboratory practice (GLP)-compliant nonclinical experiments. The results presented in this report provide definitive toxicological evidence and establish a safe dosing window for the long-term application of PFE.

## 2. Materials and Methods

### 2.1. Preparation and Characterization of PFE

The plant material was genetically identified as *P. frutescens* var. *frutescens* by matching the internal transcribed spacer (ITS, GenBank accession no. MH710687.1) and *psbA-trnH* (GenBank accession no. OR567250.1) DNA sequences with 100% identity to the NCBI GenBank database. *P. frutescens* var. *frutescens* leaves used in this study was obtained from cultivated plants at a local farm in Mangyeong-eup, Gimje-si, Republic of Korea (acquired in September 2023 and July 2024). This cultivation region consists of agricultural alluvial plains with an elevation below 50 m. The climate features warm, humid summers, with mean annual temperatures ranging from 13.7 °C to 14.6 °C and annual precipitation between 1390 mm and 1980 mm. Dried leaves of *P. frutescens* var. *frutescens* were extracted with 70% ethanol at a 1:10 (*w*/*v*) ratio for 48 h. The extract was filtered through a 1 μm filter, concentrated under reduced pressure at 65–70 °C to 10–15° Brix, and freeze-dried at −40 °C followed by vacuum drying to obtain the standardized powder (yield: 7.0%). To properly characterize the phytochemical profile and ensure the reproducibility of the study, the chemical composition of PFE was quantitatively analyzed. The analysis confirmed that the extract is rich in major phenolic and flavonoid compounds. In addition to rosmarinic acid (115.40 mg/g) as the primary marker, the extract contains significant amounts of caffeic acid (26.79 mg/g), apigenin-7-glycoside (17.37 mg/g), and luteolin-7-glycoside (3.68 mg/g). 

### 2.2. Animals

Specific pathogen-free (SPF) female Sprague–Dawley [Crl:CD(SD)] rats aged 7–8 weeks were purchased from Orient Bio, Inc. (Seongnam, Gyeonggi, Republic of Korea). The animals were housed in a facility accredited by AAALAC International and maintained in stainless steel wire cages (up to 3 rats per cage). The environmental conditions were controlled at a temperature of 22 ± 3 °C, a relative humidity of 55 ± 15%, a 12-h light/dark cycle, and an illumination intensity of 150–300 lux. A certified irradiated rodent diet (Teklad certified irradiated global 18% protein rodent diet; Inotiv, Lafayette, IN, USA) and UV-sterilized/filtered groundwater were provided ad libitum. All animals underwent an acclimation period of 7–9 days prior to the start of the study. All procedures were approved by the Institutional Animal Care and Use Committee (IACUC) of CORESTEMCHEMON, Inc. (Seongnam, Gyeonggi, Republic of Korea), a nonclinical research institute, and conducted in compliance with the Good Laboratory Practice (GLP) standards (Approval #: 2023-IA0846, 2024-IA0075, 2024-IA0632, 2024-IA0049).

### 2.3. Acute Oral Toxicity Study

The acute oral toxicity of PFE was evaluated via the acute toxic class method in accordance with the Organization for Economic Cooperation and Development (OECD) Test Guideline 423 following the application of good laboratory practice (GLP) [15]. Sequential groups of overnight-fasted female rats (*n* = 3 per step/group, total *n* = 6) received a single oral dose of PFE at 2000 mg/kg (10 mL/kg) via gavage on the basis of fasted body weight on day 1, with food restored 3–4 h post-dosing. Mortality and clinical signs were monitored continuously for the first 30 min, hourly for 4 h post-administration, and daily thereafter through Day 15. Body weights were recorded on Days 1 (predose), 2, 4, 8, and 15. On Day 15, all the animals were humanely euthanized by CO_2_ anesthesia followed by exsanguination and subjected to a thorough gross necropsy examination.

### 2.4. 28-Day Repeated-Dose Oral Toxicity Study

A repeated-dose oral toxicity study of PFE was conducted in accordance with OECD Test Guideline 407 following the application of GLP [16]. Forty rats (*n* = 10 per group; 5 males and 5 females) were assigned to four groups: a vehicle control group and three PFE treatment groups (800, 2000, and 5000 mg/kg/day). Although the standard OECD guidelines generally recommend a limit dose of 1000 mg/kg/day for chemical substances, a maximum limit dose of 5000 mg/kg/day was selected for this study. This higher limit dose is widely applied in the safety evaluation of functional food ingredients and botanical extracts to establish a robust margin of safety, considering their historically low toxicity and anticipated high human dietary exposure [17]. PFE was administered daily by oral gavage for 28 consecutive days on the basis of recent body weights. The animals were monitored daily for clinical signs and mortality. Body weights were recorded weekly and prior to necropsy, while food and water intake were measured weekly per cage. Ophthalmic examinations and urinalysis (via metabolic cages) were conducted during the final week. On day 29, following an overnight fast, the animals were anesthetized with isoflurane for blood collection from the posterior vena cava to analyze hematological (ADVIA 2120, Ozelle, Frankfurt, Germany) and clinical biochemistry (AU680, Beckman Coulter, Brea, CA, USA) parameters. Following euthanasia by exsanguination, complete gross necropsies were performed, and the absolute and relative weights of major organs (lung, liver, kidneys, spleen, heart, and thymus) were measured.

### 2.5. 90-Day Repeated-Dose Oral Toxicity Study

A repeated-dose oral toxicity study of PFE was conducted in accordance with OECD Test Guideline 408 following the application of GLP [18]. A total of 100 rats were assigned to four main groups (*n* = 20 per group; 10 males and 10 females) given vehicle control or PFE (800, 2000, and 5000 mg/kg/day) orally for 13 consecutive weeks, alongside two 28-day recovery groups (*n* = 10 per group; 5 males and 5 females for control and 5000 mg/kg/day). Mortality and clinical signs were monitored daily, and detailed clinical observations, body weights, and food/water intake data were recorded weekly. At the end of the experiment, complete gross necropsies were conducted after euthanasia by exsanguination, major organs were weighed, and hematological and clinical chemistry analyses and histopathology were performed.

### 2.6. Bacterial Reverse Mutation Assay

The bacterial reverse mutation test was conducted in accordance with OECD Test Guideline 471 using four histidine-requiring *Salmonella typhimurium* strains (TA100, TA1535, TA98, and TA1537) and one tryptophan-requiring *Escherichia coli* strain (WP2 *uvrA*) following the application of GLP [19]. Five test concentrations (50, 150, 500, 1500, and 5000 μg/plate) were selected on the basis of a preliminary range-finding study and evaluated in triplicate, both with and without metabolic activation (5% *v*/*v* rat liver S9 mix induced with phenobarbital and 5,6-benzoflavone). Using the direct plate incorporation method, 0.1 mL of test solution (or vehicle/positive control), 0.1 mL of precultured bacterial suspension (≥0.5 × 10^8^ CFU/plate), and 0.5 mL of S9 mixture (or 0.1 M sodium phosphate buffer, pH 7.4) were mixed with 2.0 mL of molten top agar and poured onto minimal glucose agar plates. Water for injection served as the negative control, whereas strain-specific mutagens (sodium azide, 2-nitrofluorene, ICR-191,4-nitroquinoline-1-oxide, 2-aminoanthracene, and benzo[a]pyrene) were used as positive controls. After incubation at 37 ± 2 °C for 50 ± 2 h, the revertant colonies were counted, and the background lawn was inspected for toxicity. A result was considered positive if a reproducible, dose-dependent or distinct increase in revertant colonies was observed in at least one strain compared with the negative control.

### 2.7. In Vitro Mammalian Chromosomal Aberration Assay

An in vitro chromosomal aberration test was conducted in Chinese hamster lung (CHL/IU) cells in accordance with OECD Test Guideline 473 following the application of GLP [20]. Exponentially growing cells (5 × 10^4^ cells/flask) were treated in duplicate across three experimental series: 6-h treatment with or without metabolic activation (2% *v*/*v* rat liver S9 mixture induced with phenobarbital and 5,6-benzoflavone), 18-h recovery, and 24-h continuous treatment without the S9 mixture. Test concentrations (125–500 μg/mL for 6 h of treatment with S9; 75–300 μg/mL for 6 h and 24 h of treatment without S9) were selected on the basis of their cytotoxicity, as evaluated by the relative increase in cell count (RICC) and maximum feasible solubility from a preliminary range-finding study. Water for injection was used as the negative control, while benzo[a]pyrene (20 mg/mL, with S9) and 4-nitroquinoline-1-oxide (0.4 mg/mL, without S9) served as positive controls. Following colcemid arrest (1 μM for 2 h) at 22 h after treatment initiation, the cells were harvested, treated with hypotonic 75 mM KCl, fixed, and stained with 5% Giemsa solution. A total of 300 metaphases per concentration (150 per replicate flask) were scored for structural and numerical aberrations under 1000× magnification.

### 2.8. Mammalian Bone Marrow Erythrocyte Micronucleus Assay

The in vivo rodent micronucleus assay was performed in accordance with OECD Test Guideline 474 following the application of GLP [21]. PFE suspended in water for injection was administered via oral gavage once daily for two consecutive days at doses of 1250, 2500, and 5000 mg/kg/day, alongside a vehicle control (10 mL/kg/day water for injection) and a positive control (40 mg/kg cyclophosphamide monohydrate, administered orally on the second dosing day). Approximately 24 h after the final treatment, bone marrow cells were harvested from femurs with fetal bovine serum, smeared onto glass slides, fixed in methanol, and stained with 0.05% acridine orange. Smears were examined under a fluorescence microscope (400× magnification) to score micronucleated polychromatic erythrocytes (MNPCEs) among 4000 PCEs per animal, while bone marrow cytotoxicity was evaluated by calculating the PCE to total red blood cell (PCE:RBC) ratio from at least 500 total erythrocytes.

### 2.9. Statistical Analysis

Statistical analyses were performed via SPSS Statistics (version 29.0; IBM Corp., Armonk, NY, USA), with a *p* value < 0.05 considered statistically significant. For in vivo repeated-dose toxicity studies and body weight/organ weight parameters, data homogeneity and normality were assessed via one-way analysis of variance (ANOVA) followed by Dunnett’s post hoc test for multiple comparisons against the vehicle control or Student’s *t* test for two-group comparisons. For the bacterial reverse mutation assay (Ames test), data were evaluated on the basis of fold increases over vehicle controls without formal statistical testing in accordance with standard guideline criteria. For the in vitro chromosomal aberration assay, the proportion of metaphases with structural or numerical aberrations was analyzed via Fisher’s exact test, and dose dependency was evaluated via the linear-by-linear association chi-square test. For the in vivo bone marrow micronucleus test, MNPCE frequencies were evaluated via the nonparametric Kruskal–Wallis H test with the post hoc Mann–Whitney U test, whereas PCE:RBC ratios were analyzed via one–way ANOVA and Student’s *t* test.

## 3. Results

### 3.1. Acute Oral Toxicity of PFE

Sequential administration of PFE at a limit dose of 2000 mg/kg body weight (*n* = 3 per step, total *n* = 6) resulted in no mortality, moribund state, or treatment-related systemic toxicity signs throughout the 14-day observation period. Although a slight, transient decrease in body weight was noted on Day 4 in two animals from the second group, normal weight gain resumed by Day 8, indicating no toxicological significance. At terminal sacrifice on day 15, gross pathological examination revealed no macroscopic lesions or abnormal changes in any organ. On the basis of these findings (zero mortality, absence of clinical toxicity, normal weight gain, and no gross pathology), PFE was classified as Category 5 or unclassified according to the GHS criteria.

### 3.2. 28-Day Repeated-Dose Oral Toxicity of PFE

#### 3.2.1. Mortality and Clinical Signs

Daily oral administration of PFE at doses up to 5000 mg/kg/day for 28 consecutive days resulted in no mortality or moribund state in male or female Sprague–Dawley rats. Treatment-related clinical observations revealed dark-brown stool (compound-colored stool) in all the animals across all the PFE-treated groups (800, 2000, and 5000 mg/kg/day) from day 2 through day 29, which showed dose-dependent intensity. Slight watery salivation was observed in high-dose (5000 mg/kg/day) animals (3/5 males from Days 15–28 and 5/5 females from Days 6–28). These clinical observations were considered nonadverse physiological responses related to test substance excretion and sensory properties (taste/smell) rather than systemic organ toxicity.

#### 3.2.2. Body Weight and Food Consumption

**Figure 1:**
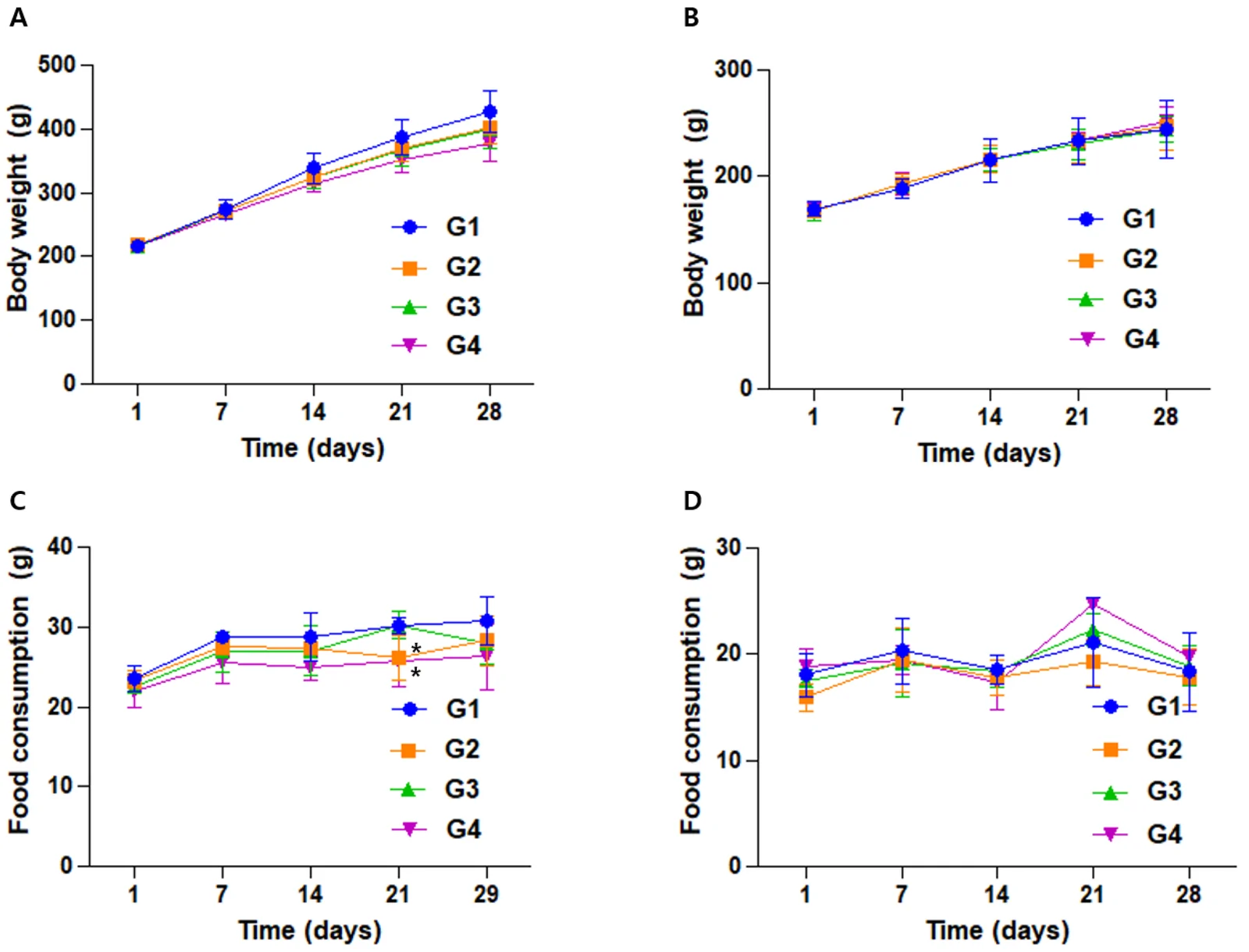
Mean body weight changes (**A**,**B**) and mean food consumption (**C**,**D**) of male and female SD rats administered PFE. Rats were treated daily with vehicle (G1) or 800 mg/kg (G2), 2000 mg/kg (G3) or 5000 mg/kg (G4) of PFE for 28 days. Data are presented as mean ± S.D. (*n* = 5 per sex) (* *p* < 0.05 vs. G1).

Compared with those of the vehicle control group, the mean daily body weights and overall body weight gain of the male rats decreased in a dose-dependent manner. On day 28, male body weight gain was reduced by 12.5%, 13.6%, and 23.1% in the 800, 2000, and 5000 mg/kg/day groups, respectively (Figure 1). However, these reductions did not reach statistical significance and remained within the facility’s historical control data range (342.74–484.68 g). Female body weight gain was unaffected by PFE treatment (Figure 1). Food consumption showed no treatment-related adverse changes in either sex, although non-dose-dependent transient changes within normal biological variations, such as decreased food intake on Day 21 in 800 and 5000 mg/kg/day in males (*p* < 0.05), were observed (Figure 1).

#### 3.2.3. Hematology and Clinical Chemistry

At the end of the experiment, hematological parameters and clinical biochemistry parameters were measured, and no treatment-related statistically significant adverse toxicological changes were observed in either male or female rats (Table 1 and Table 2). Although there was a statistically significant increase in the A/G ratio in the male 5000 mg/kg group, the total protein and albumin levels were not significantly altered (Table 2). Therefore, this biochemical fluctuation was not considered toxicologically significant.

#### 3.2.4. Organ Weights and Gross Necropsy

No significant changes in absolute organ weight were observed in either male or female rats (Supplementary Table S1). Although minorly significant increases in relative organ weight, including kidney (*p* < 0.05) and lung weights (*p* < 0.05), were observed in 5000 mg/kg-treated males (Table 3), these changes were attributed to decreased terminal body weight and were not accompanied by pathology or serum chemistry alterations. Gross necropsy at the time of sacrifice revealed no PFE-related macroscopic lesions or structural abnormalities in any organ.

### 3.3. 90-Day Repeated-Dose Oral Toxicity of PFE

#### 3.3.1. Mortality and Clinical Signs

Oral administration of PFE at doses of 800, 2000, and 5000 mg/kg/day for 90 consecutive days resulted in no mortality or moribundity in male or female Sprague–Dawley rats (*n* = 100). Treatment-related clinical observations included dark-brown stool (compound-colored stool) across all PFE-treated groups in both males and females from day 3 through the dosing period. Watery salivation was observed in high-dose (5000 mg/kg/day) animals (12/15 males from Days 2–90 and 9/15 females from Days 47–91). Both colored stool and salivation fully recovered during the 28-day treatment-free period.

#### 3.3.2. Body Weight and Food Consumption

**Figure 2:**
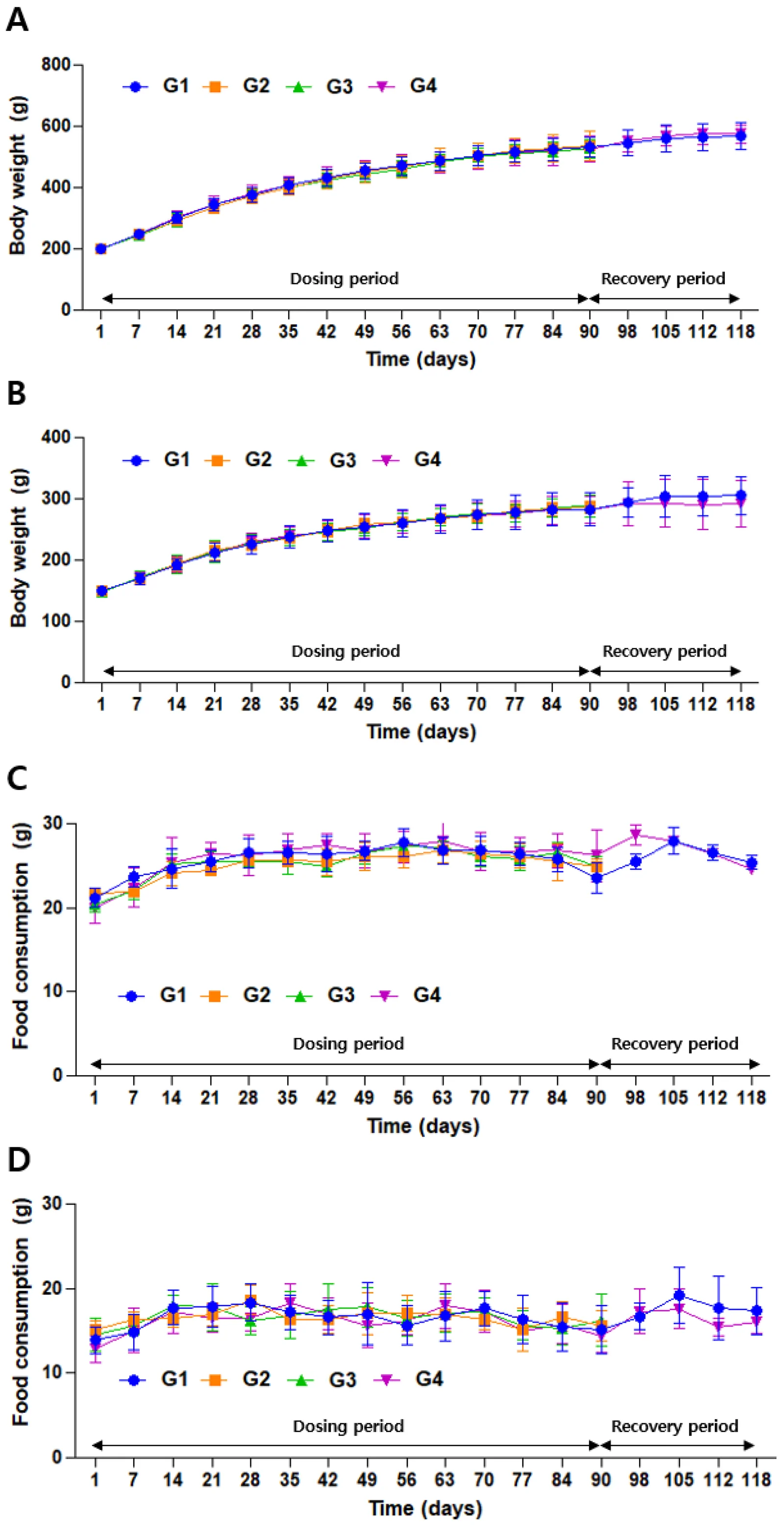
Mean body weight changes (**A**,**B**) and mean food consumption (**C**,**D**) of male and female SD rats administered PFE. Rats were treated daily with vehicle (G1) or 800 mg/kg (G2), 2000 mg/kg (G3) or 5000 mg/kg (G4) of PFE for 90 days followed by 28-day recovery period. Data are presented as mean ± S.D. (*n* = 10–15 per sex [dosing period], *n* = 5 per sex [recovery period]).

No statistically or toxicologically significant differences in mean body weights or overall body weight gains were observed between the PFE-treated groups and the vehicle control group in either sex throughout the 90-day dosing and 28-day recovery periods (Figure 2A,B). Food consumption showed minor, sporadic statistical variations that were not dose dependent and within normal historical control data ranges (Figure 2C,D, male: 11.89–30.98 g, female: 7.34–22.45 g).

#### 3.3.3. Hematology and Clinical Chemistry

At the end of the experiment, hematological parameters and clinical biochemistry parameters were measured, and test article-related but nonadverse changes were observed in both male and female rats (Table 4 and Table 5). Hematological evaluations revealed minor, dose-dependent reductions in RBC (5000 mg/kg/day in males; *p* < 0.05) and HGB/HCT (5000 mg/kg/day in males and females; *p* < 0.05) (Table 4). In terms of clinical biochemistry, significant elevations in ALP (5000 mg/kg/day in males, ≥2000 mg/kg/day in females; *p* < 0.05) and BUN (≥2000 mg/kg/day in males; *p* < 0.05) were noted (Table 5). All the clinical pathology alterations completely resolved following the 28-day recovery period and lacked histopathological correlates.

#### 3.3.4. Organ Weights and Gross Necropsy

In the main study groups, absolute and relative organ weight measurements revealed significant increases in liver and kidney weights in high-dose (5000 mg/kg/day) males and females (*p* < 0.05), as did increased lung weights (*p* < 0.05) and decreased prostate weights (with seminal vesicles/coagulating glands; *p* < 0.05) in males given 2000 mg/kg/day (Table 6 and Table 7, Supplementary Tables S2 and S3). In the recovery groups, organ weight alterations were reversed to control levels, except for a minor remaining increase in right kidney weight in 5000 mg/kg/day females (*p* < 0.05). Gross necropsy and microscopic histopathological examinations revealed no treatment-related macroscopic lesions, tissue hypertrophy, or degenerative changes in any organ, including the liver, kidneys, lungs, prostate, or thyroid gland. Minor histological findings were spontaneous or incidental, occurring at similar frequencies and severities between the control and high-dose groups.

### 3.4. Effect of PFE on Bacterial Reverse Mutation

The potential mutagenicity of PFE was evaluated in *Salmonella typhimurium* strains (TA100, TA1535, TA98, and TA1537) and *Escherichia coli* WP2 *uvrA* at concentrations up to 5000 mg/plate with and without metabolic activation (S9 mixture). Precipitation of PFE was observed at 5000 μg/plate during treatment and colony counting, but no cytotoxicity or background lawn inhibition occurred in any strain. Across all concentrations (50, 150, 500, 1500 or 5000 μg/plate), PFE produced no dose-dependent or significant increases in revertant colony counts relative to those of the vehicle controls in both the presence and absence of the S9 mixture (Table 8). Conversely, positive control mutagens produced clear, expected increases in revertant counts (3.8- to 32.3-fold increase over vehicle controls) (Table 8).

### 3.5. Effect of PFE on In Vitro Mammalian Chromosomal Aberrations

To evaluate the clastogenic and aneugenic potential of PFE (*Perilla frutescens* leaf extract), an in vitro chromosomal aberration assay was performed using Chinese hamster lung (CHL/IU) cells under three distinct treatment schedules: a 6-h exposure with or without metabolic activation followed by an 18-h recovery period and a 24-h continuous exposure without metabolic activation. Compared with vehicle control treatment, PFE treatment did not significantly or dose-dependently increase the number of metaphases with structural or numerical aberrations across all concentrations and exposure conditions (*p* > 0.05) (Table 9). The structural aberration frequencies (0.00--0.33%) remained within the historical control limits (0.00–2.00%). In contrast, the positive control mutagens produced the expected significant increases (5.33–10.00%, *p* < 0.01), confirming the sensitivity of the assay (Table 9).

### 3.6. Effect of PFE on Mammalian Bone Marrow Erythrocyte Micronucleus

The in vivo clastogenic and aneugenic potential of PFE was evaluated in male Sprague–Dawley rats administered oral doses of up to 5000 mg/kg/day for two consecutive days. No treatment-related mortality, toxicity, or significant body weight changes were observed, except for nonadverse colored stool. Bone marrow analysis at 24 h postfinal dose revealed no statistically significant or dose-dependent increases in micronucleated polychromatic erythrocytes (MNPCEs per 4000 PCEs: 0.83 ± 0.75 for the vehicle control vs. 1.83 ± 1.83, 1.67 ± 0.82, and 1.83 ± 0.98 for 1250, 2500, and 5000 mg/kg/day PFE, respectively; *p* > 0.05), with all values within historical control limits (0–4 MNPCE/4000 PCEs). The PCE:RBC ratio remained unchanged across all PFE-treated groups (0.50 ± 0.01), indicating that there was no bone marrow toxicity. Conversely, cyclophosphamide (40 mg/kg) induced a significant increase in MNPCEs (33.00 ± 5.44, *p* < 0.01) and a reduction in the PCE:RBC ratio (0.38 ± 0.02, *p* < 0.01), validating the sensitivity of the assay (Table 10).

## 4. Discussion

*Perilla frutescens* var. *frutescens* is a traditional culinary and medicinal plant with established antioxidant and anti-inflammatory properties [2,3,22]. In particular, *P. frutescens* leaf extract (PFE) has recently demonstrated significant clinical promise in improving joint function and alleviating knee discomfort in clinical settings [3]. However, despite its extensive history of dietary use and demonstrated therapeutic efficacy, the systematic nonclinical safety profile of PFE has not been previously investigated in full compliance with rigorous international regulatory guidelines. Establishing a comprehensive toxicological baseline is essential for defining a definitive safety margin for human application and supporting the commercial development of PFE as a functional dietary supplement or therapeutic agent. To address this regulatory and toxicological gap, the present study comprehensively evaluated the oral safety of PFE through a complete standard battery of nonclinical toxicity assessments, including acute oral toxicity, 28-day repeated-dose oral toxicity, a 90-day repeated-dose oral toxicity study with a 28-day recovery period, and a full three-tier genotoxicity panel, in accordance with Good Laboratory Practice (GLP) standards and OECD test guidelines.

Across all evaluated general toxicity studies, including acute (2000 mg/kg), 28-day, and 90-day repeated-dose oral studies, PFE administration produced no treatment-related mortalities, systemic toxicity signs, or adverse changes in body weight or food consumption up to the limit dose of 5000 mg/kg/day. In the 28-day repeated-dose study, a 23.1% reduction in weight gain was observed in males administered the highest dose of 5000 mg/kg/day; however, this change was not statistically significant. Beyond the lack of statistical significance, this variation is biologically interpreted as a transient, non-adverse secondary stress response rather than a manifestation of systemic toxicity. The administration of a highly concentrated PFE formulation at the maximum permissible volume (10 mL/kg) likely induced temporary feeding inefficiency and mild gastrointestinal distress due to the extract’s high viscosity and strong unpalatability. Furthermore, the relatively small sample size in the 28-day study (*n* = 5/sex) may have amplified the statistical influence of individual biological variations. Most importantly, this trend was not reproduced in the subsequent 90-day study, which utilized a larger sample cohort (*n* = 10–15/sex). The absence of any correlated adverse findings in clinical chemistry, hematology, or histopathological evaluations further substantiates that this isolated observation is not toxicologically significant. In the 90-day repeated-dose toxicity studies, the only notable findings were minor hematological and serum biochemical shifts, including dose-dependent reductions in RBC/HGB/HCT and elevations in ALP and BUN, accompanied by increased liver and kidney weights in rats administered high-dose PFE. However, comprehensive histopathological evaluations revealed no treatment-related degenerative, inflammatory, or necrotic alterations in any of the examined organs, and all the clinical pathology parameters and organ weight changes were largely reversible following the 28-day recovery period. Therefore, considering the absence of treatment-related clinical signs or histopathological changes, the limited magnitude of these changes, and their general reversibility during the recovery period, the observed hematological, clinical chemistry, and organ weight changes at 5000 mg/kg/day were considered nonadverse under the conditions of this study. Accordingly, the no observed adverse effect level (NOAEL) for PFE was considered to be 5000 mg/kg/day.

In the Ames test, PFE did not increase revertant colony frequencies across *Salmonella typhimurium* and *Escherichia coli* strains at concentrations up to the standard limit of 5000 μg/plate, both in the presence and absence of metabolic activation, indicating no potential to induce gene mutations through base–pair substitutions or frameshifts. Consistent with these findings, the in vitro chromosomal aberration assay in CHL/IU cells revealed no statistically significant or concentration-dependent increases in structural or numerical chromosomal aberrations under all short-term (with and without S9) and continuous exposure conditions, confirming the absence of clastogenic and aneugenic effects. Furthermore, in the in vivo bone marrow micronucleus assay, oral administration of PFE up to the limit dose of 5000 mg/kg/day for two consecutive days caused no increase in micronucleated polychromatic erythrocytes (MNPCEs), and the unchanged PCE:RBC ratio further demonstrated the lack of bone marrow cytotoxicity. Collectively, these concordant negative results across in vitro and in vivo systems provide no evidence of the mutagenic, clastogenic, or aneugenic activity of PFE under the conditions tested.

The safety profile of PFE observed in the present study is generally consistent with those reported for other botanical extracts evaluated in comprehensive toxicological studies. HemoHIM G, an herbal extract composed of *Angelica sinensis*, *Ligusticum chuanxiong*, and *Paeonia lactiflora*, causes no treatment-related mortality, clinical signs, or toxicologically significant changes in hematological, clinical chemistry, or histopathological parameters following repeated oral administration at doses up to 5000 mg/kg/day, and its NOAEL is considered to be 5000 mg/kg/day [22]. HemoHIM G also showed no evidence of mutagenicity or clastogenicity in a battery of in vitro and in vivo genotoxicity assays [22]. Similarly, HemoHIM, a standardized fraction derived from *Angelica gigas*, *Cnidium officinale*, and *Paeonia lactiflora*, showed no treatment-related adverse findings following 28-day or 13-week repeated oral administration at doses up to 2000 mg/kg/day, with a NOAEL of 2000 mg/kg/day and no evidence of genotoxicity [23]. In addition, fermented *Citrus sunki* peel extract showed no systemic toxicity following 90 days of oral administration at doses up to 2000 mg/kg/day and did not exhibit mutagenic, clastogenic, or micronucleus-inducing activity in the corresponding genotoxicity assays [24]. Moreover, the results of PFE presented in this report are highly consistent with the broader safety profile of the *Perilla* species reported in the literature and previous studies of extracts of *Perilla* species also demonstrated no significant oral toxicity in rodent models [25,26]. It is important to note that while the *Perilla* genus is generally safe, specific atypical chemotypes and varieties can produce potentially harmful volatile compounds. For example, perilla ketone is a known pulmonary toxin in grazing livestock, and perillaldehyde, the predominant volatile compound in *P. frutescens* var. *crispa*, has been associated with skin sensitization [27,28]. However, the specific manufacturing process of our extract, involving concentration under reduced pressure at 65–70 °C followed by vacuum freeze-drying, inherently eliminates potential volatile hazards because volatile monoterpenoids, such as perillaldehyde and perilla ketone, have high vapor pressures and are substantially vaporized and stripped away during these prolonged heating and vacuum steps. Taken together, these findings indicate that the overall safety profile of PFE, including the absence of clear treatment-related adverse effects and genotoxic potential at the tested dose levels, is comparable to that reported for other standardized or concentrated botanical extracts investigated under similar toxicological frameworks.

## 5. Conclusions

In conclusion, this study provides the first comprehensive GLP-compliant safety assessment of standardized *P. frutescens* var. *frutescens* leaf extract. PFE showed no acute oral toxicity; provided no evidence of mutagenic, clastogenic, or aneugenic activity; and demonstrated an oral NOAEL of 5000 mg/kg/day in both 28-day and 90-day repeated-dose studies. Taken together with its broad margin of safety and proven clinical joint-protective efficacy, these definitive toxicological findings support the safety of PFE for further development as a functional food or nutraceutical ingredient.

## Figures and Tables

**Table 1:** Hematological values for male and female rats in the 28-day repeated-dose oral toxicity study.

	Males				Female			
	G1	G2	G3	G4	G1	G2	G3	G4
RBC (10^6^ cells/μL)	8.0 ± 1.0	7.8 ± 0.4	8.0 ± 0.5	7.6 ± 0.2	7.6 ± 0.2	7.7 ± 0.2	7.5 ± 0.2	7.4 ± 0.3
HGB (g/dL)	15.2 ± 0.9	14.9 ± 0.2	14.9 ± 0.6	14.8 ± 0.5	14.6 ± 0.3	14.7 ± 0.3	14.5 ± 0.4	14.2 ± 0.5
HCT (%)	48.8 ± 2.6	47.3 ± 0.9	47.0 ± 1.5	47.0 ± 1.8	45.4 ± 1.3	45.5 ± 1.3	44.7 ± 1.7	44.1 ± 1.5
MCV (fL)	61.5 ± 2.1	60.5 ± 1.7	58.8 ± 2.3	61.7 ± 1.5	59.7 ± 1.1	59.5 ± 1.0	59.7 ± 1.5	59.7 ± 1.8
MCH (pg)	19.2 ± 0.5	19.0 ± 0.8	18.6 ± 0.7	19.4 ± 0.2	19.2 ± 0.4	19.2 ± 0.3	19.4 ± 0.4	19.2 ± 0.7
MCHC (g/dL)	31.2 ± 0.5	31.5 ± 0.5	31.7 ± 0.3	31.5 ± 0.5	32.2 ± 0.3	32.3 ± 0.2	32.6 ± 0.5	32.2 ± 0.3
PLT (10^3^ cells/μL)	1128 ± 120	1066 ± 107	971 ± 89	958 ± 158	1090 ± 71	1072 ± 48	1177 ± 115	1135 ± 66
WBC (10^3^ cells/μL)	11.0 ± 2.0	11.1 ± 1.8	10.2 ± 1.1	11.8 ± 2.3	6.5 ± 1.0	7.9 ± 1.8	9.0 ± 2.3	6.8 ± 1.7

The data are presented as the means ± S.Ds. (*n* = 5). Statistical significance was analyzed by one-way ANOVA and Dunnett’s *t* test.

**Table 2:** Clinical chemistry values for male and female rats in the 28-day repeated-dose oral toxicity study.

	Males				Female			
	G1	G2	G3	G4	G1	G2	G3	G4
AST (IU/L)	81.9 ± 4.9	88.5 ± 10.1	87.7 ± 10.1	87.8 ± 9.4	93.2 ± 23.2	100.2 ± 26.3	93.4 ± 14.2	91.7 ± 24.8
ALT (IU/L)	31.7 ± 3.8	31.0 ± 5.7	36.1 ± 8.4	40.7 ± 5.5	25.4 ± 2.7	23.2 ± 3.7	26.3 ± 2.8	25.7 ± 4.3
ALP (IU/L)	193.2 ± 34.4	191.8 ± 41.3	225.6 ± 17.2	210.2 ± 45.9	105.3 ± 36.7	80.9 ± 17.2	117.1 ± 19.8	150.6 ± 50.3
GLU (mg/dL)	124.6 ± 13.6	121.1 ± 12.7	119.3 ± 7.9	113.2 ± 11.7	119.8 ± 8.7	114.5 ± 19.3	109.1 ± 1.8	110.4 ± 15.0
BUN (mg/dL)	13.1 ± 0.7	11.8 ± 1.5	12.3 ± 1.7	12.2 ± 2.4	15.2 ± 1.5	16.2 ± 3.9	14.2 ± 1.0	14.9 ± 3.2
CRE (mg/dL)	0.5 ± 0.0	0.5 ± 0.1	0.5 ± 0.0	0.5 ± 0.1	0.5 ± 0.1	0.5 ± 0.1	0.5 ± 0.0	0.6 ± 0.1
TCHO (mg/dL)	57.0 ± 4.4	59.2 ± 19.2	53.4 ± 10.7	58.2 ± 16.9	61.4 ± 15.3	80.8 ± 18.3	82.6 ± 17.7	70.6 ± 19.1
TG (mg/dL)	68.4 ± 18.0	65.2 ± 42.6	51.4 ± 11.9	61.4 ± 11.1	34.2 ± 8.0	37.0 ± 11.0	35.4 ± 6.9	31.6 ± 5.3
TP (g/dL)	6.0 ± 0.2	5.8 ± 0.1	5.9 ± 0.3	5.8 ± 0.2	5.9 ± 0.4	6.3 ± 0.3	6.1 ± 0.2	6.0 ± 0.4
ALB (g/dL)	3.2 ± 0.1	3.2 ± 0.1	3.2 ± 0.1	3.2 ± 0.1	3.3 ± 0.2	3.6 ± 0.2	3.5 ± 0.1	3.4 ± 0.2
A/g ratio	1.1 ± 0.0	1.2 ± 0.0	1.2 ± 0.1	1.3 ± 0.1 *	1.3 ± 0.1	1.3 ± 0.1	1.3 ± 0.1	1.3 ± 0.1

The data are presented as the means ± S.Ds. (*n* = 5). Statistical significance was analyzed by one-way ANOVA and Dunnett’s *t* test (* *p* < 0.05 vs. G1).

**Table 3:** Relative organ weights (%) of male and female rats in the 28-day repeated-dose oral toxicity study.

	Males				Female			
	G1	G2	G3	G4	G1	G2	G3	G4
Fasting Body Weight (g)	393.0 ± 34.2	370.2 ± 22.4	363.2 ± 30.7	342.7 ± 24.7	227.8 ± 19.4	229.3 ± 17.8	228.3 ± 11.1	232.1 ± 12.5
Heart	0.33 ± 0.02	0.34 ± 0.04	0.34 ± 0.02	0.35 ± 0.01	0.40 ± 0.04	0.37 ± 0.03	0.37 ± 0.03	0.40 ± 0.03
Kidneys	0.72 ± 0.03	0.70 ± 0.09	0.72 ± 0.03	0.83 ± 0.04 *	0.74 ± 0.06	0.73 ± 0.06	0.73 ± 0.06	0.81 ± 0.05
Liver	3.08 ± 0.27	2.96 ± 0.19	2.90 ± 0.24	3.08 ± 0.11	2.96 ± 0.13	2.92 ± 0.15	3.05 ± 0.18	3.24 ± 0.24
Lung	0.37 ± 0.01	0.40 ± 0.01	0.40 ± 0.03	0.44 ± 0.01 *	0.52 ± 0.02	0.52 ± 0.04	0.53 ± 0.02	0.54 ± 0.01
Spleen	0.20 ± 0.01	0.19 ± 0.02	0.22 ± 0.04	0.20 ± 0.02	0.22 ± 0.02	0.21 ± 0.01	0.23 ± 0.03	0.23 ± 0.02
Thymus	0.13 ± 0.02	0.13 ± 0.02	0.13 ± 0.03	0.14 ± 0.02	0.18 ± 0.03	0.19 ± 0.03	0.19 ± 0.02	0.22 ± 0.04

The data are presented as the means ± S.Ds. (*n* = 5). Statistical significance was analyzed by one-way ANOVA and Dunnett’s *t* test (* *p* < 0.05 vs. G1).

**Table 4:** Hematological values for male and female rats in the 90-day repeated-dose oral toxicity study.

	Males				Female			
	G1	G2	G3	G4	G1	G2	G3	G4
RBC (10^6^ cells/μL)	8.7 ± 0.5	8.8 ± 0.4	8.7 ± 0.4	8.2 ± 0.3	7.8 ± 0.3	7.9 ± 0.5	7.8 ± 0.3	7.5 ± 0.2
HGB (g/dL)	15.3 ± 0.5	15.3 ± 0.4	15.0 ± 0.4	14.3 ± 0.4	14.4 ± 0.3	14.3 ± 0.5	14.1 ± 0.4	13.7 ± 0.4
HCT (%)	46.2 ± 1.6	46.8 ± 1.1	45.4 ± 1.1	43.6 ± 1.1	43.0 ± 1.0	42.8 ± 1.8	42.3 ± 1.4	41.2 ± 1.0
MCV (fL)	53.0 ± 2.0	53.1 ± 1.8	52.6 ± 2.3	52.9 ± 1.4	54.9 ± 1.6	54.1 ± 2.2	54.6 ± 1.5	55.0 ± 1.3
MCH (pg)	17.6 ± 1.0	17.4 ± 0.7	17.3 ± 0.8	17.4 ± 0.5	18.4 ± 0.7	18.0 ± 0.8	18.2 ± 0.7	18.3 ± 0.6
MCHC (g/dL)	33.2 ± 0.6	32.8 ± 0.4	32.9 ± 0.5	32.9 ± 0.3	33.5 ± 0.7	33.3 ± 0.4	33.2 ± 0.6	33.3 ± 0.7
PLT (10^3^ cells/μL)	997 ± 88	985 ± 42	940 ± 89	919 ± 65	1106 ± 130	973 ± 121	984 ± 110	917 ± 33
RET (%)	2.2 ± 0.3	2.2 ± 0.3	2.1 ± 0.3	2.3 ± 0.3	2.3 ± 0.6	2.3 ± 0.5	2.5 ± 0.4	2.3 ± 0.3
WBC (10^3^ cells/μL)	7.6 ± 1.4	8.1 ± 1.6	8.0 ± 1.5	7.6 ± 1.6	4.6 ± 1.2	4.2 ± 1.0	4.4 ± 1.5	4.4 ± 1.0
NEU (%)	18.9 ± 7.1	15.7 ± 5.5	16.1 ± 3.0	17.7 ± 3.1	13.8 ± 6.1	14.7 ± 3.4	14.4 ± 3.7	14.1 ± 4.2
LYM (%)	74.8 ± 7.2	77.8 ± 5.9	76.9 ± 4.2	75.8 ± 2.6	79.6 ± 6.1	77.7 ± 3.8	79.0 ± 3.9	80.0 ± 4.1
MONO (%)	3.5 ± 0.7	3.4 ± 0.8	3.5 ± 1.1	3.5 ± 0.7	3.2 ± 0.9	4.1 ± 1.0	3.2 ± 0.6	2.9 ± 0.5
EOS (%)	1.5 ± 0.8	1.8 ± 0.6	2.2 ± 1.0	1.6 ± 0.6	1.8 ± 0.5	2.2 ± 0.8	2.3 ± 1.0	1.8 ± 0.7
BASO (%)	0.1 ± 0.1	0.2 ± 0.1	0.1 ± 0.1	0.1 ± 0.0	0.1 ± 0.1	0.1 ± 0.1	0.1 ± 0.1	0.1 ± 0.1
PT (Sec)	8.5 ± 0.2	8.5 ± 0.3	8.4 ± 0.2	8.2 ± 0.2	7.9 ± 0.2	7.9 ± 0.1	7.8 ± 0.1	7.8 ± 0.1
APTT (Sec)	17.1 ± 0.9	17.4 ± 1.2	16.5 ± 0.9	16.3 ± 0.8	15.2 ± 0.9	15.6 ± 0.9	15.2 ± 1.6	14.9 ± 1.1

The data are presented as the means ± S.Ds. (*n* = 5). Statistical significance was analyzed by one-way ANOVA and Dunnett’s *t* test.

**Table 5:** Clinical chemistry values for male and female rats in the 90-day repeated-dose oral toxicity study.

	Males				Female			
	G1	G2	G3	G4	G1	G2	G3	G4
AST (U/L)	73.4 ± 17.4	67.1 ± 10.2	70.2 ± 9.5	67.2 ± 10.1	79.6 ± 21.5	75.3 ± 17.6	73.3 ± 20.3	63.0 ± 9.5
ALT (U/L)	28.3 ± 4.9	29.8 ± 5.1	29.8 ± 3.1	33.8 ± 6.5	24.7 ± 3.7	27.5 ± 6.5	22.5 ± 2.0	24.1 ± 4.3
ALP (U/L)	76.4 ± 20.6	96.5 ± 12.3	102.9 ± 36.3	129.2 ± 25.6	41.5 ± 9.5	60.8 ± 12.5	63.0 ± 23.0	61.9 ± 22.0
CPK (IU/L)	147.2 ± 196.2	101.0 ± 48.1	102.1 ± 59.1	122.8 ± 86.4	138.4 ± 112.2	112.7 ± 69.5	148.3 ± 191.1	83.9 ± 77.1
GLU (mg/dL)	138.6 ± 14.5	150.3 ± 26.1	145.4 ± 15.4	132.8 ± 17.8	118.8 ± 11.4	119.8 ± 13.4	121.9 ± 15.8	116.2 ± 15.9
BUN (mg/dL)	12.8 ± 1.0	13.3 ± 1.4	14.9 ± 2.5	16.7 ± 1.8	17.2 ± 2.6	15.7 ± 1.7	17.5 ± 2.5	18.3 ± 2.4
CRE (mg/dL)	0.5 ± 0.1	0.5 ± 0.0	0.5 ± 0.0	0.5 ± 0.0	0.5 ± 0.0	0.5 ± 0.1	0.5 ± 0.1	0.6 ± 0.1
TBIL (mg/dL)	0.16 ± 0.01	0.17 ± 0.02	0.16 ± 0.01	0.17 ± 0.02	0.19 ± 0.03	0.18 ± 0.03	0.18 ± 0.02	0.19 ± 0.02
TCHO (mg/dL)	78.1 ± 20.5	67.6 ± 10.5	76.1 ± 14.2	69.5 ± 11.3	72.5 ± 17.5	69.6 ± 18.0	75.6 ± 19.8	80.9 ± 15.7
TG (mg/dL)	75.7 ± 13.9	93.5 ± 27.5	74.0 ± 22.7	74.3 ± 36.6	35.1 ± 6.5	38.1 ± 8.8	37.7 ± 6.0	37.6 ± 9.1
TP (g/dL)	6.0 ± 0.3	6.1 ± 0.3	6.1 ± 0.2	6.1 ± 0.2	5.9 ± 0.3	5.9 ± 0.2	5.9 ± 0.3	5.9 ± 0.3
ALB (g/dL)	3.0 ± 0.1	3.1 ± 0.1	3.1 ± 0.1	3.1 ± 0.1	3.2 ± 0.2	3.1 ± 0.1	3.1 ± 0.2	3.2 ± 0.2
A/g ratio	1.0 ± 0.0	1.0 ± 0.1	1.0 ± 0.1	1.0 ± 0.1	1.2 ± 0.1	1.1 ± 0.0	1.1 ± 0.1	1.2 ± 0.1
IP (mg/dL)	6.0 ± 0.6	5.9 ± 0.4	5.9 ± 0.3	6.5 ± 0.5	5.0 ± 0.9	5.2 ± 0.6	5.1 ± 0.6	5.3 ± 0.6
Ca (mg/dL)	9.7 ± 0.3	9.9 ± 0.3	9.9 ± 0.2	9.9 ± 0.2	9.6 ± 0.3	9.6 ± 0.2	9.6 ± 0.3	9.9 ± 0.3
Na (mmol/L)	141.4 ± 0.8	141.0 ± 1.3	140.5 ± 1.4	138.4 ± 1.0	139.6 ± 1.1	139.8 ± 1.3	139.2 ± 1.0	138.2 ± 1.3
K (mmol/L)	4.2 ± 0.3	4.1 ± 0.2	4.2 ± 0.2	4.0 ± 0.2	4.1 ± 0.3	4.0 ± 0.2	4.0 ± 0.3	3.8 ± 0.1
LDL (mg/dL)	5.7 ± 1.7	4.5 ± 1.3	4.5 ± 0.9	5.5 ± 1.8	4.2 ± 1.3	4.3 ± 1.5	3.9 ± 0.6	5.0 ± 1.4
HDL (mg/dL)	25.1 ± 4.6	23.7 ± 2.9	26.1 ± 2.8	26.3 ± 3.3	24.9 ± 3.6	24.4 ± 4.9	26.7 ± 5.8	28.3 ± 3.9

The data are presented as the means ± S.Ds. (*n* = 5). Statistical significance was analyzed by one-way ANOVA and Dunnett’s *t* test.

**Table 6:** Relative organ weights (%) of male rats in the 90-day repeated-dose oral toxicity study.

	G1	G2	G3	G4
Fasting Body Weight (g)	505.07 ± 31.03	506.17 ± 40.81	492.86 ± 31.52	476.63 ± 42.92
Adrenal glands	0.0105 ± 0.0011	0.0102 ± 0.0016	0.0105 ± 0.0017	0.0107 ± 0.0011
Brain	0.4410 ± 0.0202	0.4406 ± 0.0195	0.4403 ± 0.0393	0.4555 ± 0.0383
Epididymis	0.2910 ± 0.0355	0.2926 ± 0.0360	0.2958 ± 0.0331	0.3173 ± 0.0332
Heart	0.2906 ± 0.0168	0.2828 ± 0.0183	0.2975 ± 0.0232	0.3019 ± 0.0158
Kidneys	0.5845 ± 0.0531	0.5866 ± 0.0318	0.6211 ± 0.0360	0.7205 ± 0.0504 *
Liver	2.6723 ± 0.2521	2.6858 ± 0.1849	2.8660 ± 0.1951	3.2782 ± 0.1848 *
Lung	0.3510 ± 0.0233	0.3543 ± 0.0287	0.3750 ± 0.0203	0.4066 ± 0.0276 *
Pituitary gland	0.0026 ± 0.0003	0.0024 ± 0.0002	0.0025 ± 0.0003	0.0026 ± 0.0003
SV-CG and Prostate Gland	0.7389 ± 0.0475	0.6778 ± 0.0659	0.6884 ± 0.0605	0.6672 ± 0.0907
Spleen	0.1813 ± 0.0346	0.1744 ± 0.02687	0.1760 ± 0.0257	0.1718 ± 0.0306
Testis	0.8285 ± 0.1011	0.8416 ± 0.0843	0.8583 ± 0.0882	0.8819 ± 0.0723
Thymus	0.0658 ± 0.0192	0.0623 ± 0.01131	0.0588 ± 0.0133	0.0525 ± 0.0112
Thyroid gland w/parathyroid gland	0.0046 ± 0.0008	0.0043 ± 0.0008	0.0042 ± 0.0007	0.0043 ± 0.0004

The data are presented as the means ± S.Ds. (*n* = 5). Statistical significance was analyzed by one-way ANOVA and Dunnett’s *t* test (* *p* < 0.05 vs. G1).

**Table 7:** Relative organ weights (%) of female rats in the 90-day repeated-dose oral toxicity study.

	G1	G2	G3	G4
Fasting Body Weight (g)	263.18 ± 26.13	268.75 ± 9.24	269.35 ± 15.91	261.61 ± 14.28
Adrenal glands	0.0251 ± 0.0048	0.0260 ± 0.0023	0.0257 ± 0.0029	0.0257 ± 0.0027
Brain	0.7614 ± 0.0685	0.7492 ± 0.0427	0.7311 ± 0.0467	0.7709 ± 0.0445
Heart	0.3328 ± 0.0255	0.3294 ± 0.0111	0.3405 ± 0.0236	0.3319 ± 0.0156
Kidneys	0.6183 ± 0.0537	0.6292 ± 0.0551	0.6441 ± 0.0583	0.7269 ± 0.0507 *
Liver	2.5361 ± 0.3076	2.5804 ± 0.1826	2.8533 ± 0.3726	3.1042 ± 0.3095 *
Lung	0.4887 ± 0.0235	0.4863 ± 0.0295	0.5022 ± 0.0212	0.5497 ± 0.0300 *
Pituitary gland	0.0053 ± 0.0006	0.0051 ± 0.0009	0.0055 ± 0.0005	0.0056 ± 0.0004
Spleen	0.2166 ± 0.0267	0.2259 ± 0.0213	0.2169 ± 0.0288	0.2155 ± 0.0244
Ovaries	0.0313 ± 0.0054	0.0310 ± 0.0047	0.0321 ± 0.0062	0.0291 ± 0.0035
Thymus	0.0837 ± 0.0112	0.0825 ± 0.0169	0.0868 ± 0.0108	0.0874 ± 0.0125
Thyroid gland w/parathyroid gland	0.0066 ± 0.0014	0.0062 ± 0.008	0.0063 ± 0.0015	0.0064 ± 0.0013
Uterus & cervix	0.2732 ± 0.0889	0.2972 ± 0.0757	0.3767 ± 0.1375	0.3669 ± 0.1512

The data are presented as the means ± S.Ds. (*n* = 5). Statistical significance was analyzed by one-way ANOVA and Dunnett’s *t* test (* *p* < 0.05 vs. G1).

**Table 8:** Bacterial reverse mutation test results for PFE in *S. typhimurium* and *E. coli* strains with and without metabolic activation.

Strain	Test Substance	Dose (μg/plate)	With S9 Mix	Without S9 Mix
TA98	PFE	0	34 ± 3	32 ± 4
		50	31 ± 3	32 ± 4
		150	33 ± 3	27 ± 2
		500	28 ± 4	32 ± 3
		1500	25 ± 4	24 ± 2
		5000	35 ± 1	33 ± 2
	Benzo[a]pyrene	1	129 ± 6	-
	2-Nitrofluorene	2	-	306 ± 13
TA100		0	105 ± 6	101 ± 6
		50	105 ± 5	106 ± 5
		150	100 ± 2	101 ± 2
		500	110 ± 6	100 ± 2
		1500	111 ± 7	108 ± 6
		5000	117 ± 2	107 ± 7
	2-aminoanthracene	1	2973 ± 138	-
	Sodium azide	0.5	-	618 ± 21
TA1535		0	18 ± 3	18 ± 1
		50	15 ± 3	16 ± 3
		150	14 ± 2	18 ± 3
		500	12 ± 1	18 ± 3
		1500	14 ± 1	15 ± 2
		5000	14 ± 1	15 ± 2
	2-aminoanthracene	2	403 ± 16	-
	Sodium azide	0.5	-	571 ± 25
TA1537		0	16 ± 2	10 ± 1
		50	16 ± 3	12 ± 1
		150	15 ± 3	8 ± 2
		500	17 ± 1	8 ± 2
		1500	13 ± 2	12 ± 3
		5000	16 ± 3	12 ± 1
	2-aminoanthracene	1	448 ± 11	-
	Acridine mutagen ICR 191		-	120 ± 8
WP2 *uvrA*		0	20 ± 3	18 ± 1
		50	22 ± 1	20 ± 3
		150	23 ± 2	20 ± 3
		500	22 ± 4	17 ± 2
		1500	22 ± 4	15 ± 3
		5000	23 ± 1	21 ± 4
	2-aminoanthracene	6	145 ± 4	-
	4-nitroquinoline	0.5	-	115 ± 7

The data are presented as the means ± S.Ds. (*n* = 3).

**Table 9:** In vitro chromosomal aberration assay results for PFE in CHL/IU cells.

Test Substance	Conc. (μg/mL)	S9 Mix	Exposure-Recovery (h)	Metaphases Analyzed	RICC (%)	Number of Structural Aberrations	Number of Numerical Aberrations
Vehicle	0	-	6–18	300	100	0	0
PFE	75	-	6–18	300	92	0	0
PFE	150	-	6–18	300	67	0	0
PFE	200	-	6–18	300	61	1	0
PFE	300	-	6–18	300	41	0	0
4-nitroquinoline	0.4	-	6–18	300	71	30	0
Vehicle	0	+	6–18	300	100	0	0
PFE	125	+	6–18	300	97	1	0
PFE	250	+	6–18	300	81	0	0
PFE	300	+	6–18	300	58	1	0
PFE	500	+	6–18	300	42	0	0
Benzo[a]pyrene	20	+	6–18	300	72	17	0
Vehicle	0	-	24–0	300	100	0	0
PFE	75	-	24–0	300	88	0	0
PFE	150	-	24–0	300	81	0	0
PFE	200	-	24–0	300	53	0	0
PFE	300	-	24–0	300	40	0	0
4-nitroquinoline	0.4	-	24–0	300	70	16	0

Statistical significance was analyzed by Fisher’s exact test and linear-by-linear association Chi-square test. RICC: Relative Increase in Cell Number.

**Table 10:** Results for PFE in male SD rats in mammalian bone marrow erythrocyte micronucleus test.

Test Substance	Dose (mg/kg/day)	Number of Animals	MNPCE/4000 PCEs	PCE:RBC Ratio	% of Vehicle Control
Vehicle Control (Water)	0	6	0.83 ± 0.75	0.50 ± 0.01	100
PFE	1250	6	1.83 ± 1.83	0.50 ± 0.01	99
PFE	2500	6	1.67 ± 0.82	0.50 ± 0.02	99
PFE	5000	6	1.83 ± 0.98	0.50 ± 0.01	99
Cyclophosphamide	40	6	33 ± 5.44	0.38 ± 0.02	75

Data are presented as mean ± S.D. (*n* = 6). Statistical significance was analyzed by non-parametric Kruskal–Wallis H-test with post hoc Mann–Whitney U-test for MNPCE and One-way ANOVA and Students *t*-test for PCE:RBC ratio.

## Data Availability

The original contributions presented in this study are included in the article/Supplementary Materials. Further inquiries can be directed to the corresponding authors.

## Supplementary Materials

The following supporting information can be downloaded at https://www.mdpi.com/article/10.3390/foods15213852/s1, Table S1: Absolute organ weight (g) of male and female rats in 28-day repeated dose oral toxicity test; Table S2: Absolute organ weight (g) of male rats in 90-day repeated dose oral toxicity study; Table S3: Absolute organ weight (g) of female rats in 90-day repeated dose oral toxicity study.

## Abbreviations

The following abbreviations are used in this manuscript:

RBC	Red Blood Cells
HGB	Hemoglobin
HCT	Hematocrit
MCV	Mean Corpuscular Volume
MCH	Mean Corpuscular Hemoglobin
MCHC	Mean Corpuscular Hemoglobin Concentration
PLT	Platelets
WBC	White Blood Cells
RET	Reticulocytes
NEU	Neutrophils
LYM	Lymphocytes
MONO	Monocytes
EOS	Eosinophils
BASO	Basophils
PT	Prothrombin Time
APTT	Activated Partial Thromboplastin Time
AST	Aspartate Aminotransferase
ALT	Alanine Aminotransferase
ALP	Alkaline Phosphatase
GLU	Glucose
BUN	Blood Urea Nitrogen
CRE	Creatinine
TCHO	Total Cholesterol
TG	Triglycerides
TP	Total Protein
ALB	Albumin
A/g ratio	Albumin/Globulin Ratio
IP	Inorganic Phosphate
LDL	Low-Density Lipoprotein
HDL	High-Density Lipoprotein
