# Supplementary material for: Comprehensive Toxicological Evaluation and Safety Assessment of Perilla frutescens Leaf Extract: Repeated-Dose Oral Toxicity and Genotoxicity Studies
Source: Foods. 2026 Oct 28;15(21):3852. doi: 10.3390/foods15213852 (PMC13763472; doi:10.3390/foods15213852)
Supplement: Supplementary file 1 [file foods-15-03852-s001.zip › foods-4613121-supplementary.pdf]

Table S1. Absolute organ weight (g) of male and female rats in 28-day repeated dose oral toxicity test.

	Males				Female			
	G1	G2	G3	G4	G1	G2	G3	G4
Heart	1.29 ± 0.10	1.25 ± 0.10	1.23 ± 0.07	1.19 ± 0.07	0.92 ± 0.10	0.84 ± 0.05	0.83 ± 0.06	0.94 ± 0.09
Kidneys	2.84 ± 0.24	2.59 ± 0.18	2.62 ± 0.28	2.85 ± 0.30	1.68 ± 0.14	1.67 ± 0.15	1.67 ± 0.21	1.88 ± 0.18
Liver	12.16 ± 2.10	10.98 ± 1.27	10.58 ± 1.64	10.56 ± 1.15	6.76 ± 0.71	6.70 ± 0.66	6.97 ± 0.54	7.54 ± 0.93
Lung	1.46 ± 0.11	1.49 ± 0.07	1.45 ± 0.13	1.52 ± 0.13	1.20 ± 0.08	1.19 ± 0.05	1.21 ± 0.09	1.26 ± 0.07
Spleen	0.78 ± 0.12	0.71 ± 0.08	0.79 ± 0.17	0.70 ± 0.12	0.50 ± 0.09	0.48 ± 0.04	0.52 ± 0.08	0.53 ± 0.04
Thymus	0.53 ± 0.12	0.48 ± 0.07	0.47 ± 0.11	0.47 ± 0.05	0.40 ± 0.07	0.44 ± 0.06	0.44 ± 0.03	0.51 ± 0.09

Data are presented as mean ± S.D. (n=5). Statistical significance was analyzed by one-way ANOVA and Dunnett's *t*-test (* $p < 0.05$ vs. G1).

Table S2. Absolute organ weight (g) of male rats in 90-day repeated dose oral toxicity study.

	G1	G2	G3	G4
Adrenal glands	0.0527 ± 0.0058	0.0516 ± 0.0066	0.0513 ± 0.0083	0.0508 ± 0.0081
Brain	2.2227 ± 0.0724	2.2267 ± 0.1568	2.1609 ± 0.0980	2.1588 ± 0.1170
Epididymis	1.4638 ± 0.1465	1.4723 ± 0.1392	1.4518 ± 0.1237	1.5039 ± 0.1245
Heart	1.4664 ± 0.1088	1.4302 ± 0.1400	1.4631 ± 0.1102	1.4390 ± 0.1480
Kidneys	2.9486 ± 0.2825	2.9665 ± 0.2514	3.0613 ± 0.2656	3.4277 ± 0.3159*
Liver	13.4792 ± 1.3339	13.6066 ± 1.5479	14.0935 ± 0.8731	15.6157 ± 1.5920*
Lung	1.7686 ± 0.0942	1.7925 ± 0.1958	1.8464 ± 0.1295	1.9319 ± 0.1399
Pituitary gland	0.0130 ± 0.0015	0.0121 ± 0.0013	0.0124 ± 0.0016	0.0123 ± 0.0017
SV-CG and Prostate Gland	3.7245 ± 0.2221	3.4129 ± 0.2172	3.3862 ± 0.2863*	3.1679 ± 0.4087*
Spleen	0.9092 ± 0.1296	0.8828 ± 0.1484	0.8627 ± 0.0966	0.8173 ± 0.1495
Testis	4.1658 ± 0.3711	4.241 ± 0.3432	4.2226 ± 0.4482	4.189 ± 0.3485

Thymus	0.3307 ± 0.0889	0.3168 ± 0.0706	0.2878 ± 0.0598	0.2512 ± 0.0642
Thyroid gland w/ parathyroid gland	0.0232 ± 0.0036	0.0214 ± 0.0035	0.0207 ± 0.003	0.0203 ± 0.003

Data are presented as mean ± S.D. (n=5). Statistical significance was analyzed by one-way ANOVA and Dunnett's *t*-test (* $p < 0.05$ vs. G1).

Table S3. Absolute organ weight (g) of female rats in 90-day repeated dose oral toxicity study.

	G1	G2	G3	G4
Adrenal glands	0.0653 ± 0.0113	0.0698 ± 0.0060	0.0692 ± 0.0095	0.0674 ± 0.0092
Brain	1.9891 ± 0.0767	2.0117 ± 0.1006	1.9635 ± 0.0654	2.0119 ± 0.0688
Heart	0.8732 ± 0.0828	0.8848 ± 0.0291	0.9158 ± 0.0649	0.8683 ± 0.0637
Kidneys	1.6267 ± 0.2226	1.6881 ± 0.1196	1.7322 ± 0.1583	1.9010 ± 0.1581*
Liver	6.6842 ± 1.1983	6.9311 ± 0.4957	7.6518 ± 0.7581	8.1279 ± 0.9977*
Lung	1.2847 ± 0.1269	1.3066 ± 0.0837	1.3511 ± 0.0700	1.4351 ± 0.0542*
Pituitary gland	0.0140 ± 0.0017	0.0138 ± 0.0025	0.0147 ± 0.0019	0.0148 ± 0.0015
Spleen	0.5730 ± 0.1119	0.6063 ± 0.0492	0.5840 ± 0.0838	0.5635 ± 0.0672
Ovaries	0.0827 ± 0.0173	0.0833 ± 0.0137	0.0868 ± 0.0189	0.0761 ± 0.0105
Thymus	0.2214 ± 0.0453	0.2216 ± 0.0454	0.2343 ± 0.0374	0.2283 ± 0.0332
Thyroid gland w/	0.0174 ± 0.0036	0.0167 ± 0.0023	0.0170 ± 0.0038	0.0167 ± 0.0032

parathyroid gland				
Uterus & cervix	0.7136 ± 0.2178	0.7957 ± 0.1863	1.0092 ± 0.3442	0.9569 ± 0.3905

Data are presented as mean ± S.D. (n=5). Statistical significance was analyzed by one-way ANOVA and Dunnett's *t*-test (* $p < 0.05$ vs. G1)
